# A Novel *EMD* Mutation Identified by Whole-Exome Sequencing in Twins with Emery–Dreifuss Muscular Dystrophy

**DOI:** 10.1155/2020/2071738

**Published:** 2020-08-24

**Authors:** Xiafei Dai, Rong Luo, Yang Chen, Chenqing Zheng, Yibin Tang, Hongmei Zhang, Ye Su, Tao He, Xiaoping Li

**Affiliations:** ^1^School of Medicine, University of Electronic Science and Technology of China, Chengdu, Sichuan 610054, China; ^2^Department of Cardiology, Sichuan Academy of Medical Sciences and Sichuan Provincial People's Hospital, Chengdu, Sichuan 610072, China; ^3^Institute of Cardiovascular Disease, Chengdu Medical College, Chengdu, Sichuan 610500, China; ^4^Shenzhen RealOmics (Biotech) Co., Ltd., Shenzhen 518081, China

## Abstract

This case reports a novel hemizygous frameshift *EMD* mutation (c.487delA, p.Ser163fs) in twins of an Emery–Dreifuss muscular dystrophy family with severe cardiac involvement and mild muscle weakness. Their mother carried the same heterozygous mutation.

## 1. Introduction

Emery–Dreifuss muscular dystrophy (EDMD) is a rare genetic disease with an estimated incidence of 3/1,000,000 [1]. EDMD is clinically characterized by a triad of (1) slowly progressive scapulo-humero-peroneal muscle weakness at childhood, (2) early joint contractures of the elbow flexors, Achilles tendons, and neck extensors, and (3) cardiomyopathy with conduction block, arrhythmia, such as atrial flutter, which may result in sudden death [2–5]. Genetically, at least six genes (*EMD*, *LMNA, FHL1, TMEM43, SYNE1*, and *SYNE2*) are associated with EDMD, where *EMD* and *FHL1* are the most familiar [6, 7]. To date, 357 mutations have been identified in the *EMD* gene (https://www.ncbi.nlm.nih.gov/clinvar/). The *EMD* gene consists of six exons, which are located at Xq28 and encode a protein termed emerin [8]. Emerin contains three domains of a hydrophilic nucleoplasmic domain, a transmembrane region, and a short C-terminal tail [9]. Emerin is a ubiquitously expressed protein in skeletal and cardiac muscle, and it plays an important role in gene expression, cell signaling, and protein-protein interactions.

Herein, we report here a novel hemizygous frameshift *EMD* mutation (c.487delA, p.Ser163fs) in twins of an EDMD family with severe cardiac involvement and mild muscle weakness.

## 2. Material and Methods

**Figure 1:**
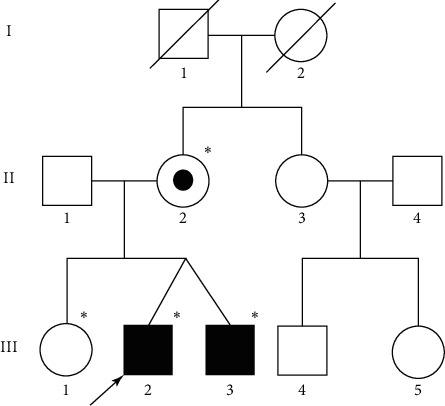
Pedigree structure. Square: male; circle: female; black square: affected; white circle/square: unaffected; white circle/square with a black dot: carrier. The arrow indicates the proband. ^*∗*^Sequenced family members.

Four subjects (Figure 1, II-2, III-1, III-2, and III-3) in a family from Sichuan Province of China were enrolled in our study. This study was approved by the Ethics Committee of the Sichuan Academy of Medical Sciences and Sichuan Provincial People's Hospital. Written informed consent was obtained from each of the participants. The medical history of the pedigree was recorded in detail. Whole-exome sequencing (WES) was performed in the proband.

### 2.1. Whole-Exome Sequencing and Sanger Sequencing Validation

Qualified genomic DNA of a proband (Figure 1) was hybridized with the AIExomeV1 58 M enrichment kit (iGeneTech, Beijing, China) to enrich exonic DNA for constructing a sequencing library. We then performed sequencing on the Illumina Xten platform (Illumina, Shanghai, China) with paired-end 150 bp readings independently for the captured library to ensure that this sample had average coverage of 137-fold.

### 2.2. Data Preprocessing

Samples were aligned to the NCBI human genome reference assembly (hg19) using Burrows–Wheeler Aligner. We then used Picard MarkDuplicates (http://broadinstitute.github.io/picard/) to mark the duplicate readings to mitigate biases introduced by data generation, such as PCR amplification. Binary Alignment Map (BAM) files were processed using the Genome Analysis Toolkit (GATK v3.7; https://software.broadinstitute.org/gatk/) to perform realignment around known indels. We then recalibrated the base quality scores for the individual base calls in each sequence reading.

### 2.3. Discovery of Variants

Germline short variant discovery was carried out using analysis-ready BAM files, and it produced variant calls. GATK (v3.7) HaplotypeCaller was used to call variants per sample in targeted and flanking regions for each individual to produce a file in genome variant call file (GVCF) format. We then performed joint genotyping to combine the multisample GVCF. We used genotype GVCFs to obtain multisample genotypes for all sites. Finally, a hard filter was applied for filtering to produce the final multisample call set with the desired balance of precision and sensitivity.

### 2.4. Annotation

SnpEff (http://snpeff.sourceforge.net) was used to separate single-nucleotide variants into different functional categories according to their gene location and their expected effect on encoded gene products, based on information from the reference sequence database. All variants were further annotated by the control population of the 1000 Genomes Project (October 2014 release, http://www.1000genomes.org), ExAC (https://exac.broadinstitute.org), EVS (http://evs.gs.washington.edu/EVS), the disease databases of ClinVar (http://www.ncbi.nlm.nih.gov/clinvar), and OMIM (http://www.omim.org).

### 2.5. Case Report

**Figure 2:**
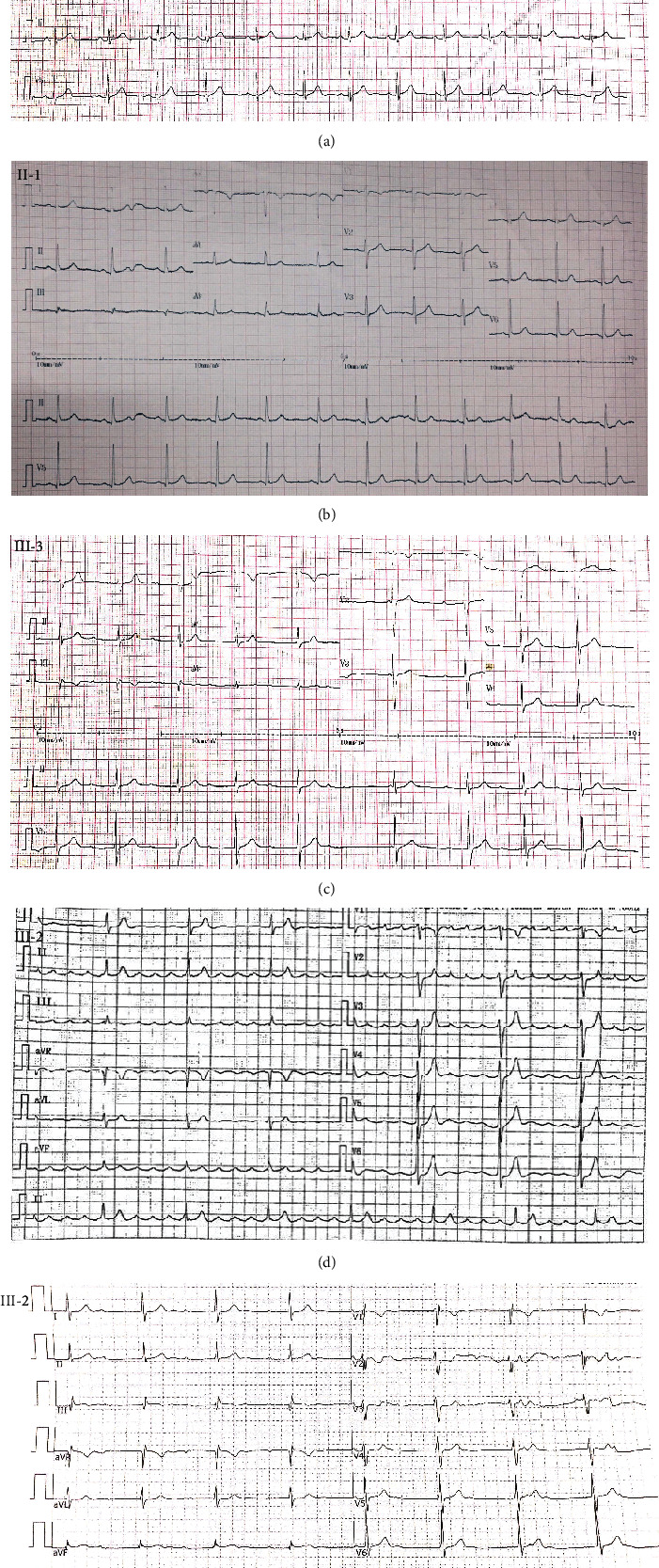
ECGs of the pedigree (II-2, III-1, III-2, and III-3). (a) III-1 was normal. (b) II-2 was normal. (c) III-3 showed second-degree atrioventricular block and bradycardia. (d) An ECG of III-2 showed atrial flutter and third-degree atrioventricular block before the operation. (e) An ECG of III-2 showed a junctional escape rhythm, third-degree atrioventricular block.

**Figure 3:**
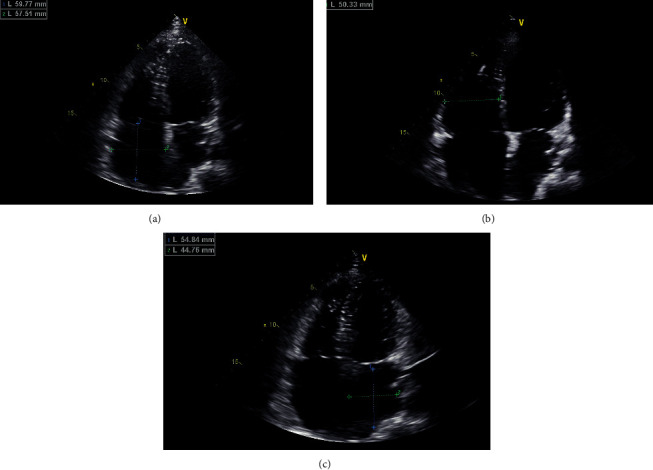
Echocardiography of the proband. (a) Right atrium (59 × 57 mm). (b) Right ventricle (50 mm). (c) Left atrium (54 × 44 mm).

The proband was the elder brother of the twins, aged 23 years, and he was admitted to our hospital with palpitation. Both of the twins had difficulty in straightening their elbows since birth and no muscle weakness and spine involvement according to their description. The proband had a suspicious history of viral myocarditis at the age of 7 years, and he then received radiofrequency ablation for atrial flutter in our hospital at the age of 23 (Figure 2(d)). After the operation, he recovered with junctional escape rhythm, third-degree atrioventricular block (Figure 2(e)). But the proband refused to receive pacemaker implantation and medication. Echocardiography imaging showed enlargement of the right atrium (59 × 50 mm), right ventricle (46 mm), and left ventricle (58 mm). Laboratory tests showed that levels of creatinine kinase (685U/L, normal range: 50–310 U/L), creatinine kinase-MB (25.3 ng/ml, normal range: 0–6.6 ng/ml), and myoglobin (283.3 ng/ml, normal range: 0–140.1 ng/ml) were elevated. Postoperation echocardiography of the proband is shown in Figure 3. An electrocardiogram (ECG) of the proband's brother showed second-degree atrioventricular block (Figure 2(c)). ECGs of the sister and mother were normal (Figures 2(a) and 2(b)). Before genetic testing, the proband was misdiagnosed with dilated cardiomyopathy.

## 3. Results

**Figure 4:**
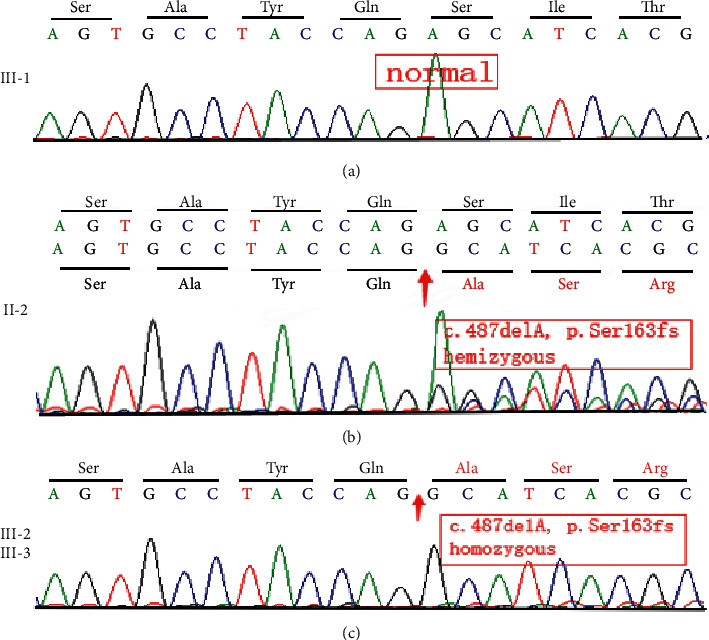
Sanger sequencing DNA chromatogram of the pedigree (II-2, III-1, III-2, and III-3). (a) III-1 was normal. (b) II-2 presented with a heterozygous frameshift mutation (c.487delA, p.Ser163fs). (c) III-2 and III-3 showed a hemizygous frameshift mutation (c.487delA, p.Ser163fs).

Mutation analysis showed a novel hemizygous frameshift mutation (NM_000117.2:c.487delA [p.Ser163fs]) in exon 6 of the *EMD* gene in the twins. This mutation was validated by Sanger sequencing. Family aggregation analyses showed that the twin's mother carried the same heterozygous mutation, but she was asymptomatic. This finding indicated that this mutation was X-linked inherited. This mutation was not detected in other family members (Figure 4).

## 4. Discussion

In this study, we identified a novel hemizygous deletion (c.487delA) in exon 6 of the *EMD* gene in twins suffering from EDMD. This mutation, we speculated, may result in a frameshift and a premature stop codon after amino acid 163 (p.Ser163fs) according to the reference. To the best of our knowledge, this mutation is novel and has not been reported yet.

The EMD gene is 2100 bp in length and is located in Xq28 [9]. This gene has six exons encoding a ubiquitous protein termed emerin, which contains 254 amino acids. Emerin plays important roles in maintaining nuclear structure, function, and stability via interaction with other inner nuclear membrane proteins, such as lamina-associated polypeptide, lamin, and B receptor, particularly in muscle [10–13]. In the heart, emerin protein is specifically situated at desmosomes and fasciae adherentes. Mutations in *EMD* might lead to truncated emerin and damage and degeneration of myocytes. The clinical manifestations of EDMD are variable, which might be associated with the position of mutations. Shigehisa et al. [7] reported two probands with *EMD* gene mutations (c.153_154insC in exon 2 and c.359_362delCAGT in exon 4) who presented with a diverse mild echocardiogram and ECG. Recently, Zhou et al. [14] reported a novel frameshift mutation (c.253_254insT, p.Y85Lfs ^*∗*^ 8) in exon 3 of *EMD*, where the proband showed generalized cardiomegaly. A splice acceptor site mutation, c.450-2A > G (p.Arg150fs), was identified in the *EMD* gene of another patient who presented with no cardiomegaly, as reported by Yuan et al. [9]. In addition, according to the CA Brown et al. [6] and Nevo et al. [15], mutations in exon 2 in *EMD* is a hotspot. Typical pathogenic variant types in *EMD* are point mutations and small deletions.

In this study, the consequences of a frameshift mutation (p.Ser163fs) in the proband were encoding of a truncated emerin protein with 163 amino acids. This caused the absence of the transmembrane region and a short C-terminal tail. A lack of emerin in the heart might change myocardial cell adhesion and electrophysiology, which could contribute to cardiac conduction block [16]. Clinically, our patient presented with mild joint contracture and muscle weakness. However, he had serious cardiac involvement with atrial flutter, sick sinus syndrome, high-degree atrioventricular conduction block, and enlargement of the right ventricle, right atrium, and left atrium. These findings are different from those in the aforementioned studies. Therefore, we speculate that the novel mutation in our patient had a more severe effect on cardiac than skeletal muscle [17–19].

Interestingly, because of this novel frameshift mutation (p.Ser163fs), the elder twin had a more severe phenotype than the younger twin. The elder twin presented with enlargement of the heart, mainly the right ventricle and right atrium, atrial flutter and third-degree atrioventricular block, and a junctional escape rhythm after the operation. However, the younger twin only showed second-degree atrioventricular block in an ECG. Both of them had mild joint contracture and muscle weakness. This discrepancy in the phenotype might be due to individual heterogeneity and incomplete penetrance of the mutation. Environmental deviation should also be taken into consideration. We conclude that the clinical manifestations were disparate, even with the same mutation. Additionally, the same heterozygous mutation was detected in the twin's mother without any symptoms, which indicated that EDMD was inherited in the form of X-linked recession. Female carriers of this mutation should be aware of the risk of developing lethal cardiac conduction defects [15, 20, 21].

In conclusion, a novel frameshift mutation, which was identified in twins, has expanded the spectrum of EMD gene mutations and provided a more accurate diagnosis clinically. Because the twins in our study presented with various clinical presentations, we conclude that the phenotype of EDMD is different, even with the same mutation. Therefore, follow-up for the pedigree is necessary.

## Data Availability

No data were used to support this study.
